# Case Report: A report on the countermeasures after PICC line breakage in 3 postoperative breast cancer patients

**DOI:** 10.3389/fsurg.2024.1416855

**Published:** 2024-08-20

**Authors:** Yuqin Jing, Jing Qu, Shuo Jiang, Xiaomin Wang

**Affiliations:** ^1^ Breast Surgery, Jinan Maternity and Child Care Hospital Affiliated to Shandong First Medical University, Jinan, China; ^2^ Department of Nursing, Jinan Maternity and Child Care Hospital Affiliated to Shandong First Medical University, Jinan, China

**Keywords:** breast cancer, peripherally inserted central catheter, breakage, countermeasures, case report

## Abstract

Peripherally inserted central catheter (PICC) is a widely used technique in oncology chemotherapy, characterized by safety, reliability, and long dwell time. However, a catheter can break due to various causes. When an acute rupture occurs, it always lead to sever complications which may threaten patients’ safety and potentially result in medical disputes. In this study, we collected and analyzed 3 cases of PICC line breakage causing drug leakage in our hospital from 2018 to 2023. All these 3 cases were postoperative breast cancer patients accepting chemotherapy, with 2 cases involving external partial breakage and 1 case involving internal partial breakage. Due to timely and appropriate management, no acute rupture occurred. We propose some ideas such as selecting high-quality catheter materials and avoiding over extension or repeated bending are crucial in preventing PICC line breakage. In addition, we also recommend establishing a standardized and scientific management pattern of PICC to ensure the safety and effectiveness of its clinical application, including comprehensive assessment, “four-element” intervention, and continuous evaluation.

## Introduction

Chemotherapy is the main and commonly used treatment for breast cancer, and intravenous infusion is its main method of administration (1). Traditional intravenous infusion requires repeatedly inserting the infusion needle into the vein. However, chemotherapy drugs are highly irritating with severe toxic side effects, and the chemotherapy cycles for malignant tumors such as breast cancer are long. Traditional intravenous infusion not only increases the patients’ suffering but also adds to the workload of nursing staff (2). Peripherally inserted central catheter (PICC) not only reduces the pain caused by repeatedly inserting but also effectively avoids vascular damage caused by long-term infusion of irritating drugs. Its application in clinical settings, especially during chemotherapy for malignant tumor patients, is becoming increasingly widespread (3).

PICC refers to a catheterization technique that enters the central venous system through peripheral veins (such as the cephalic vein, median cubital vein, and basilic vein), with the catheter tip reaching the cavoatrial junction (4). After successful placement, maintenance is required once a week, involving procedures like dressing changes, flushing, etc., with a duration of approximately one year. PICC is characterized by minimal trauma, ease of operation, long retention time, and avoidance of drug extravasation. It is widely used in fields such as parenteral nutrition, chemotherapy, and antibiotic therapy, providing a safe and reliable intravenous infusion channel for patients (5). However, with prolonged catheterization, various complications may occur, especially catheter rupture. The ruptured end of the catheter may drift with the blood flow to the heart or pulmonary artery, causing serious complications such as pulmonary embolism and arrhythmias, potentially endangering life (6). Literature reports a rupture rate of PICC catheters ranging from 0.67% to 3.5% (4). Since the introduction of PICC technology in our hospital in 2018, 252 cases have been successfully catheterized, with 3 cases of catheter breakage occurring (a rate of 1.19%). Among them, there was 1 case of external partial breakage and 2 cases of internal partial breakage, none of which led to severe acute complete rupture. Due to timely detection and appropriate management, no harm was caused to the patients. The following describes the process and nursing experiences in response to these incidents.

## Case presentation

A review of 3 cases of PICC line breakage in the breast surgery ward of Jinan Maternal and Child Health Hospital from 2018 to 2023 was conducted. Patients’ age, PICC batch number, catheter insertion site, puncture times, dwelling length, exposed length, damage location, PICC tip positioning, maintenance times, cause of breakage, treatment measures, and prognosis were summarized and analyzed. All 3 patients had successful and uneventful first-time insertions, with no adverse reactions during the indwelling period. Case 1 and case 2 had catheters placed in the left upper limb (PICC batch numbers REGN0292), maintained for 19 and 7 times respectively. After 125 and 50 days of indwelling respectively, intracorporeal partial breakages occurred (dwelling lengths were 40 cm and 44.5 cm, PICC tip positions at T6 and T7, damage locations at 39.8 cm and 44.2 cm). Case 3 had a catheter placed in the right upper limb (PICC batch number REFT2318), maintained for 19 times. After 132 days of indwelling, extracorporeal partial breakage occurred (catheter dwelling length 39 cm, PICC position at T7, damage location at 39.5 cm) (Table 1).

**Table 1:** Information of 3 patients with PICC line breakage.

Patient number	Age (year)	Insertion site	Dwelling length (cm)	Exposed length (cm)	Tip position	Rupture position(cm)	Puncture times / Smoothly?	Batch number	Maintenance times
1	40	Left upper limb	40	7	T6	39.8	1/ Yes	REGN0292	19
2	42	Left upper limb	44.5	7	T7	44.2	1/ Yes	REGN0292	7
3	50	right upper limb	39	7	T7	39.5	1/ Yes	REFT2318	19

## Case 1

The patient was admitted on October 16th, 2023 for the 6th cycle of chemotherapy following breast cancer surgery. Observation revealed no change in the dwelling length of the catheter or arm circumference, no redness or swelling in the surrounding skin, no leakage or tenderness at the insertion site, no abnormalities in the dressing, and the catheter was functioning well.

On October 17th, the PICC line was flushed before chemotherapy, and the process was normal. About 20 min after the chemotherapy drug was administered, approximately 0.2 ml of milky white fluid was found to be leaking from the PICC insertion site. There was no pain or redness in the surrounding skin. Suspecting damage to the catheter at some point, the nurse clamped the fluid infusion immediately, used sterile gauze to absorb leaked liquid and cleaned the surrounding skin with saline solution. Afterwards, it was discovered the fluid is leaking from the inside of puncture site after multiple flushes (the insertion point scale at 40 cm). To further investigate the cause, the catheter was pulled out by 1 cm. During the subsequent flushing, water beads were found at 39.8 cm, indicating damage to the catheter at that point leading to fluid leakage. After informing the patient of the risk of line breakage and treatment plan, the nurse withdrew the catheter to 34 cm and trimmed with 5 cm exposed.

A chest x-ray showed the tip of the PICC line reaching the upper edge of the 4th thoracic vertebra, deviating from its normal position. To ensure the patient's safety, the nurse removed the catheter after the completion of this chemotherapy cycle.

## Case 2

The patient was admitted on October 23th, 2023 for the 3rd cycle of chemotherapy after breast cancer surgery. Observation revealed no change in the dwelling length of the catheter or arm circumference, no redness or swelling in the surrounding skin, no leakage or tenderness at the puncture site, no abnormalities in the dressing, and the catheter was functioning well.

We flushed the PICC line before chemotherapy on October 24th, and the process was normal. However, approximately 0.5 ml of clear fluid was found to be leaking from the PICC insertion site about 15 min after infusion with no pain or redness in the surrounding skin. Suspecting damage to the catheter at some point, immediate measures were taken to avoid the acute rupture, including clamping the fluid infusion, absorbing the leakage with sterile gauze and rinsing the surrounding skin with saline solution. After multiple flushes, we discovered the fluid was leaking from the needle eye (the insertion point scale at 44.5 cm).

To further investigate the cause, we withdrew the catheter by 1 cm. Upon further flushing, it was observed that there was a water droplet formation at 44.2 cm, indicating a possible damage at that point causing the leakage. After informing the patient of the risk of line breakage and treatment plan, we withdrew the catheter to 39 cm and trimmed it with 5 cm exposed.

The chest x-ray result revealed the presence of the PICC line shadow, with its tip positioned at the upper edge of the 4th thoracic vertebra, deviating from the normal position. To ensure the patient's safety, we removed the catheter after the completion of this chemotherapy cycle.

## Case 3

The patient was admitted on October 23th, 2023 for the 6rd cycle of chemotherapy after breast cancer surgery. Observation revealed no change in the dwelling length of the catheter or arm circumference, no redness or swelling in the surrounding skin, no leakage or tenderness at the puncture site, no abnormalities in the dressing, and the catheter was functioning well.

On October 24th, we flushed the catheter before chemotherapy, and the process was normal. About 20 min after the intravenous infusion of glutathione, approximately 0.5 ml of clear fluid was found to be seeping from the PICC insertion site without any pain or redness on the surrounding skin. Suspecting a break in the catheter, the fluid infusion was immediately clamped and sterile gauze was used to absorb the leaked fluid. After multiple flushes, we observed that there was water droplet-like leakage forming on the exterior part of the line (0.5 cm away from the insertion point), confirming a break at this point leading to fluid leakage. We informed the patient of the treatment plan and withdrew the line to 34 cm and trimmed it with 5 cm exposed.

A chest x-ray revealed the positioning of the PICC line, with its tip located above the right upper edge of the 4th thoracic vertebra, slightly deviating from the normal position. To ensure the patient's safety, the nurse removed the catheter after the completion of this chemotherapy cycle.

## Discussion

In the 1990s, PICC technology was introduced in China. Due to its advantages such as small trauma, easy operation, long retention time, and avoiding drug extravasation, it is now widely used in clinical practice (7). For breast cancer patients, clinical treatment usually focuses on surgical treatment supplemented by chemotherapy to effectively control the systemic spread of malignant tumors and prevent postoperative recurrence and metastasis (8). Due to the often lengthy chemotherapy cycles and the strong acid or alkali characteristics of chemotherapy drugs, blood vessels are susceptible to drug damage, leading to problems such as peripheral venous inflammation and drug extravasation. Therefore, the higher safety factor of PICC is often used in clinical practice (9).

Considering that breast cancer patients may experience complications such as lymphedema in the affected limb postoperatively, the catheter is generally placed in the healthy limb to avoid such issues. However, this can potentially cause frequent bending or even breakage to the catheter due to patients’ excessive reliance on the healthy limb for daily activities (10). In addition, rough handling during catheter maintenance, long-term exposure to medications and disinfectants, as well as the material of the catheter itself, are also factors that cannot be ignored in causing catheter damage (11).

Since the introduction of PICC technology in our hospital in 2018, 252 catheterizations have been successfully performed with 3 cases of catheter breakage occurring (an incidence rate of 1.19%). Among these cases, 2 involved internal partial breakage and 1 involved external partial breakage, with none being severe cases of acute complete rupture. Experience include: (1) Standardized training. Regular training and assessment can improve the puncture skills of PICC specialist nurses, ensuring that the first operation is smooth with a success rate of 100%. (2) Strict monitoring. Especially during chemotherapy infusion, nurses observe patients’ condition closely and inquire about their complaints with regular checks every 15–30 min. (3) Promptly dispose. Nurses are able to identify signs of catheter breakage in a timely manner, such as leakage at the puncture site, catheter dislodgement, etc., and promptly deal with them according to the emergency procedures for PICC line rupture. For example, immediate ECG monitoring should be performed to detect early abnormal rhythm, and chest x-ray examination should also be conducted as soon as possible to better prevent complications related to internal rupture through chest x-ray images. However, in order to investigate the causes of the catheter breakage, a review and analysis were conducted by interviewing medical staffs, patients and their family members, as well as the manufacturer, respectively.

1. Catheter factors: The catheters used in the 3 cases are Bard three-way valve catheters made of silicone material. Silicone has poor toughness and is prone to damage and cracking. Relevant studies (12, 13) have shown that different chemotherapy solutions have no significant effect on the degradation of polyurethane and silicone materials. However, as the implantation time increases, the differences in mechanical properties between different materials become more pronounced. The mechanical properties of polyurethane materials do not show a significant decrease, while silicone materials exhibit an increase in mechanical test unevenness with the increase of implantation time. Currently, in many Western hospitals, silicone catheters have been completely abandoned and replaced with PICC catheters made of higher-grade materials such as polyurethane. Clinical use has shown that the new generation of polyurethane PICC catheters have better biocompatibility, higher tensile strength, become softer upon entering the body, and have stronger resistance to chemical drugs. Therefore, the only reasonable “strategy” to effectively reduce PICC catheter breakage is to avoid using silicone catheters. However, considering that in most developing countries including China, different populations are constrained by economic conditions, and the influence of medical insurance policies, it is currently not possible to fully popularize the use of higher-grade material PICC catheters, especially in grassroots areas. Based on this, it is suggested that the government, under the current level of medical care and socio-economic development, should promptly achieve the update of PICC catheter materials and include more higher-grade PICC catheters within the scope of medical insurance policy support to reduce the burden on patients and increase clinical popularization and usage.
2. Patient factors: On one hand, patients receiving long-term chemotherapy may have vascular spasms caused by the strong irritative effects of the drugs, leading to damage to the PICC line (14). On the other hand, subjective non-compliance of patients can be influenced by objective conditions (15). The 3 patients in this report were all housewives with poor financial conditions and unable to afford the expenses of hiring a maid. High workload in daily household chores leaded to frequent and significant excessive movements of the limbs with indwelling catheters. Relevant literature (16) reports that excessive frequent bending of the limb on the punctured side causes repeated folding at the junction of the catheter and connector, resulting in wear of the silicone catheter's inner membrane, leading to catheter damage or breakage. This explains why these 3 catheter breakages all occurred near the insertion site.

Based on the above analysis, standardized and normalized scientific management model should be established for the management of PICC placement in postoperative breast cancer patients, including assessment, intervention, and evaluation as three continuous and complete processes. Firstly, comprehensive assessment. Healthcare professionals should comprehensively assess various indicators of PICC placement patients, fully anticipate the limitations in various factors of environmental, social psychological, physiological, and health-related behavior domains (17). Before carrying out interventions for specific patients, focus should be placed on potential issues the patients may have, timely supplementing other disease-related issues, emphasizing personalized education and guidance work, and strengthening self-management compliance and enthusiasm. Secondly, the “Four-element” intervention. Considering the insufficient professional nursing resources for post-discharge care of breast cancer postoperative patients with PICC, exploration should be made into establishing a multidisciplinary collaborative team led by a nursing referral specialist, utilizing “Internet + nursing” services (18), constructing systemic, universal, and operational discharge plan projects, transitioning from nursing within the hospital to long-term continued care for patients post-discharge (19). A PICC management record should be established during the patient's hospitalization, and it should be referred to the community (or other medical institutions) through a nursing referral specialist before discharge; after discharge, with the help of the “PICC maintenance network”, continuous assessment and educational guidance of patient and family self-management abilities should be provided by the nursing referral specialist, as well as coordination with community or other medical institution medical staffs, ensuring continuous information, management, and care-patient relationship for post-discharge care (20). By implementing the “four-element linkage” continuity care management model of “hospital-community-family-patient”, the management of PICC for postoperative breast cancer patients can be effectively improved. Finally, continuous evaluation. To ensure the safety and effectiveness of PICC, an continuous evaluation plan should be implemented for patients and their family caregivers, including aspects such as skin assessment, catheter position evaluation, catheter function assessment, medication administration evaluation, catheter infection assessment, patient self-assessment, etc (21). Based on the evaluation results, continuous improvement should be made to comprehensively address the complex needs of patients after discharge, reduce the incidence of catheter breakage or rupture, and further enhance the clinical application effectiveness of PICC technique.

## Data Availability

The original contributions presented in the study are included in the article/Supplementary Material, further inquiries can be directed to the corresponding author.
